# Inhibitory Effect of (*2R*)-1-(1-Benzofuran-2-yl)-N-propylpentan-2-amine on Lung Adenocarcinoma

**DOI:** 10.1007/s12253-019-00603-6

**Published:** 2019-02-08

**Authors:** Zsolt Mervai, Andrea Reszegi, Ildikó Miklya, József Knoll, Zsuzsa Schaff, Ilona Kovalszky, Kornélia Baghy

**Affiliations:** 1 1st Department of Pathology and Experimental Cancer Research, Semmelweis University, Üllői út 26., Budapest, H-1085 Hungary; 2 Department of Pharmacology and Pharmacotherapy, Semmelweis University, Budapest, Hungary; 3 2nd Department of Pathology, Semmelweis University, Budapest, Hungary

**Keywords:** BPAP, Lung adenocarcinoma, Cancer, Tumor inhibition, FVB/N, Geroconversion

## Abstract

BPAP is a potent enhancer substance with catecholaminergic and serotoninergic activity in the brain. It was discovered that it is also effective against certain types of experimental cancers, showing the most promising results in case of lung cancer. That is why we tested its efficacy in two different doses in a newly developed EGFR wild type mouse lung adenocarcinoma xenograft model. Experiments were conducted on FVB/N and SCID mouse strains treated with low and high dose of BPAP. Body weight, survival, and tumor volumes were recorded. Furthermore, the activity of major signaling pathways of NSCLC such as MAPK and Akt/mTOR as well as cell cycle regulation were determined. Significant inhibition of tumor growth was exerted by both doses, but the mechanism of action was different. High dose directly inhibited, whereas low dose activated the main signaling pathways. Exposure to low dose BPAP resulted in elevated activity of the mTOR pathway together with p16^INK^-induced cell cycle arrest, a typical feature of geroconversion, a senescent state characterized by loss of cell proliferation. Finally the events culminated in cell cycle inhibition point in case of both doses mirrored by the decrease of cyclin D1, CDK4 and PCNA. In addition, BPAP treatment had a beneficial effect on bodyweight suggesting that the compound at least in part is able to compensate the cancer-related wasting. In view of the low toxicity and confirmed antitumor effect of BPAP against experimental lung adenocarcinoma, this novel compound deserves further attention.

**Electronic supplementary material:**

The online version of this article (10.1007/s12253-019-00603-6) contains supplementary material, which is available to authorized users.

## Introduction

The phenethylamine derivative (−)-deprenyl (also known as selegiline) gained attention in the 1970s when its ability to block MAO-B enzyme [1] was confirmed and was hence the first to be used in the treatment of Parkinson’s disease [2]. Longevity studies showed that deprenyl can prolong the lifespan of rats, mice and beagle dogs when given in a daily dose of 0.25 mg/kg [3–5]. Later on 1-phenyl-2-propylaminopentane (PPAP) was synthetized, lacking the MAO inhibitory effect but still being a potent enhancer of catecholaminergic signaling in the central nervous system [6]. Based on above results, a tryptamine derived new compound, (2R)-1-(1-Benzofuran-2-yl)-N-propylpentan-2-amine (BPAP) was created, capable of potentiating catecholaminergic and serotonergic activity in the brain [7]. BPAP has been shown to have neuroprotective and memory stimulatory effects similar to those of deprenyl but without the ability to inhibit the MAO-B enzyme [8].

Both BPAP and deprenyl are capable of exerting their effects in a very low concentration range [9]. The impact on learning skills and lifespan of long-term deprenyl and BPAP treatment at low concentration ranges was monitored in a longevity study carried out on Wistar rats. Both molecules exhibited a lifespan-extending effect, but BPAP showed more promising results [10]. It was noticed that significantly less spontaneous fibrosarcomas developed in the treated groups than in the control group [11]. Based on these observations, we tested the effects of low-dose BPAP treatment in different tumor models.

In an in vivo model of colon cancer with liver metastasis, BPAP significantly reduced the number of metastasis [11]. In addition, BPAP was tested in other tumor models as well: xenograft models of mouse colon carcinoma and lung adenocarcinoma and in a primary hepatocarcinogenesis experiment induced by diethylnitrosamine in mice. The most promising results were obtained in the lung tumor model established from a FVB/N strain, in which lung adenocarcinomas developed after exposure to single doses of diethylnitrosamine [12].

Lung cancer is the most common cancer in Europe and the United States claiming the largest number of lives [13, 14]. Histologically, the disease is divided into two major classes: small cell lung cancer (SCLC) and non-small cell lung cancer (NSCLC) [15]. The latter is the more common and in the United States 85% of lung cancers are NSCLC [15]. In the last decade, adenocarcinomas became the dominant representative within NSCLC [16].

It is not unprecedented that a molecule effective in a non-malignant disorder is found to inhibit certain types of cancers [17]. Several nonsteroidal anti-inflammatory drugs, statins and other types of anti-psychotic drugs were shown to have anti-tumor effect [17].

In the present work, we aimed to examine the effects and mechanism of action of BPAP in lung adenocarcinomas.

## Materials and Methods

### Animals and Treatments

All animal experiments were conducted according to the ethical standards of the Animal Health Care and Control Institute of Csongrád County, Hungary and the protocol was approved by their Committee (permit No. XVI/03047–2/2008). Animals were obtained from the animal facility of the 1st Department of Pathology and Experimental Cancer Research of Semmelweis University (Budapest, Hungary).

To examine the effects of BPAP on lung adenocarcinoma, a subcutaneous mouse tumor model was applied [12]. To this end, a total of 45 FVB/N male mice at the age of 8 weeks were utilized, 27 in Experiment 1 and 18 in Experiment 2. Animals were treated on a daily basis with 0.0001 mg/kg (low dose) or 0.05 mg/kg (high dose) BPAP starting from the day after subcutaneous tumor inoculation. The control group received saline once every day. Tumors were measured 3 times a week by the same investigator using a digital caliper. Tumor volume was calculated using the following formula: V (mm^3^) = (width^2^ (mm) × length (mm) × π)/6. Mice were terminated by cervical dislocation in ether anesthesia. Tumors were removed, their weight measured, were stored at −80 °C. The experiment was repeated on SCID mice to determine whether the adaptive immune system has a role in the tumor suppressor effect of BPAP (Experiment 3). Eighteen animals were utilized in this 3rd study, and were terminated after 28 days.

### Materials

Deprenyl was supplied by Sanofi-Chinoin (Budapest, Hungary) and BPAP by Fujimoto Pharmaceutical Corporation (Osaka, Japan).

### Western Blot

Frozen tumor tissues were homogenized and suspended with lysis buffer (containing: 20 mM Tris pH = 7.5, 150 mM NaCl, 2 mM EDTA, 0.05% Triton X-100, 0.5% Protease Inhibitor Cocktail (P8340, Sigma-Aldrich, St. Louis, MO), 2 mM Na_3_VO_4_ and 10 mM NaF). Samples were sonicated and kept on ice before centrifugation at 13000 rpm at 4 °C for 10 min. Protein concentrations were measured by Bradford method [18]. Thirty μg total proteins were mixed with loading buffer containing β-mercaptoethanol and denatured at 99 °C for 5 min.

Denatured samples were loaded onto a 10% SDS-polyacrylamide gel and separated for 40 min at 200 V. Proteins were transferred to a PVDF membrane with overnight blotting at 4 °C at a constant 75 mA. Successful blotting was confirmed by Ponceau staining. Blocking procedure was carried out with 5% non-fat dry milk dissolved in TBS. The primary antibodies applied with their appropriate dilutions are listed in **Supplementary Table** **1**. For negative control, 1% non-fat dry milk (in TBS) without primary antibody was applied. Incubation was overnight at 4 °C with gentle shaking. Secondary antibody was anti-rabbit or anti-mouse polyclonal HRP-conjugated antibody (Dako, Glostrup, Denmark) dissolved in 1% non-fat dry milk (in TBS) applied for 1 h at room temperature with gentle shaking. For washing 0.05% Tween20 in TBS was used for 5 × 5 minutes between each step. For detection, SuperSignal West Pico Chemiluminescent Substrate (34,078, Thermo Fischer Scientific, Waltham, MA) was applied. Bands were detected with Kodak Image Station 4000 mm (Kodak, Rochester, NY). The bands were evaluated with GelAnalyzer program.

### Statistical Analysis

All statistical analyses were performed using GraphPad Prism 7.00 software (Graphpad Software Inc., La Jolla, CA, USA). Data were tested for normal distribution using the omnibus normality test of D’Agostino & Pearson. Significance of changes were tested using a nonparametric test (the Mann–Whitney U-test) or the Student’s t test, depending on the distribution of the data. The independent experimental sets were compared for reproducibility. Only reproducible significant changes were considered as significant. Significance was declared at the standard *p* < 0.05 level.

## Results

### BPAP Inhibits Tumor Growth

**Fig. 1:**
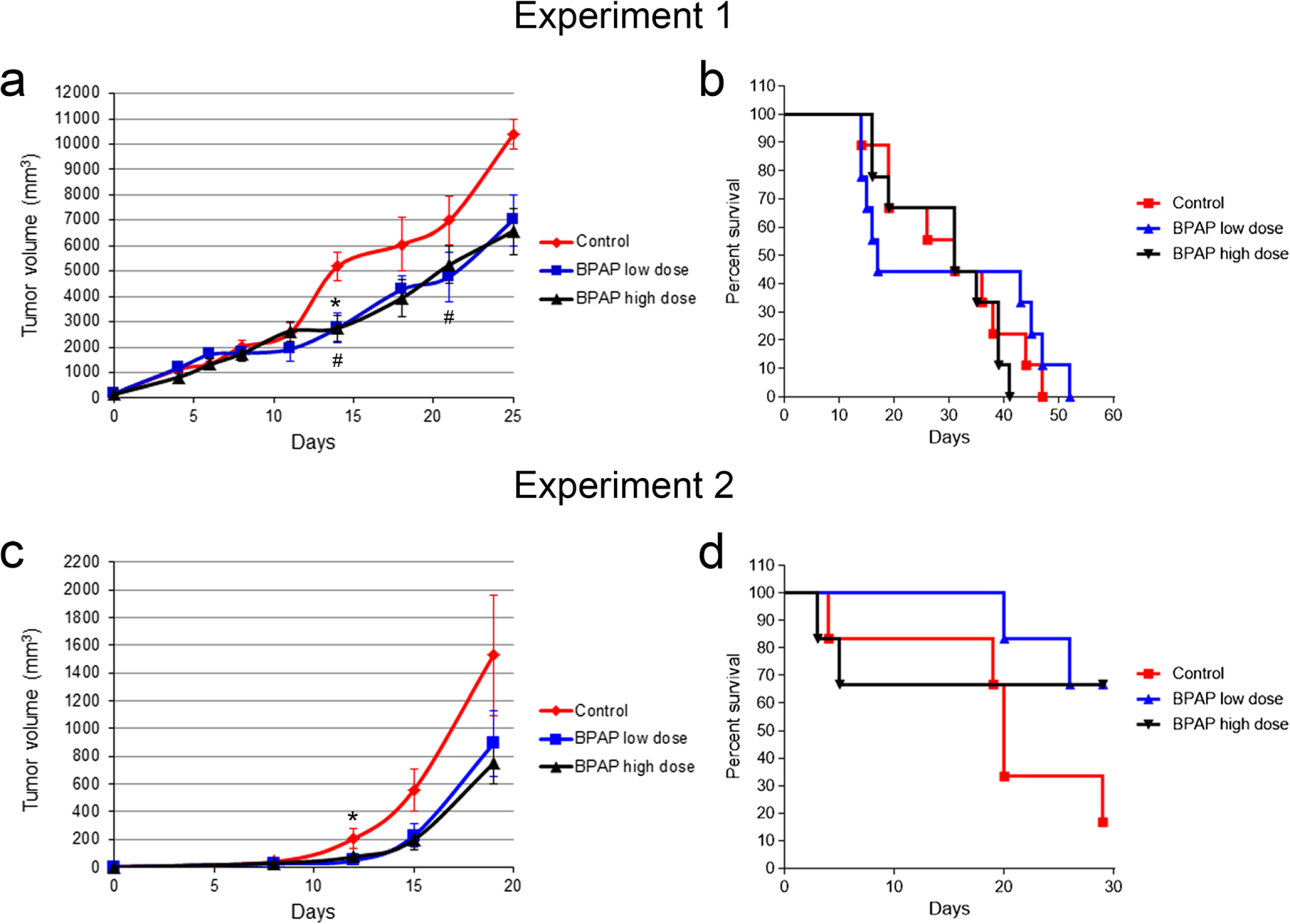
**Growth curve of subcutaneous lung tumor and survival of FVB/N mice.** Two independent experiments were performed. Changes in tumor volume **(a)** and survival rate of the animals **(b)** in Experiment 1. Changes in tumor volume **(c)** and survival rate **(d)** in Experiment 2. The data are the mean ± SE of the individual groups. *P < 0.05 for BPAP low dose vs. control; #P < 0.05 for BPAP high dose vs. control

The effect of BPAP on tumor growth was studied in 3 independent experiments. Experiment 1 was performed on FVB/N mice using our subcutaneous lung cancer xenograft model [12], where both low and high doses of BPAP significantly inhibited tumor growth (*p* < 0.05) (Fig. 1**a**). Here, the treatments had only minor effect on the survival of animals (Fig. 1b). In Experiment 2, the entire study was repeated with smaller inoculated tumors aiming to detect changes in both tumor growth and survival of animals. Indeed, BPAP treatment in both doses resulted in reduced tumor size compared to control (Fig. 1c). In harmony with the previous study, tumor volumes decreased with ~40% and ~50% after BPAP low- and high-dose treatment, respectively (*p* < 0.05) (Fig. 1c). In addition, BPAP exposure extended the survival of animals compared to control ones (Fig. 1d).

**Fig. 2:**
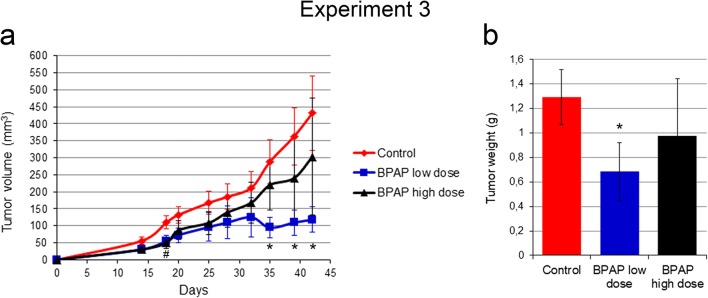
**Growth curve of subcutaneous lung tumor and tumor volume in SCID mice.** Temporal changes of tumor volume **(a)**, and the tumor weight **(b)** at the end of the experiment. The data are the mean ± SE of the individual groups. *P < 0.05 for BPAP low dose vs. control; #P < 0.05 for BPAP high dose vs. control

The experiment was repeated again in a SCID mouse strain to see whether the adaptive immune system plays a role in the promotion of the anti-tumor effect of BPAP (Experiment 3). In harmony with the previous experiments, BPAP treatment inhibited tumor growth at both concentrations (Fig. 2a). Low-dose BPAP reduced the tumor size by ~40%, the high-dose by ~25% (*p* < 0.05), respectively (Fig. 2b).

### Changes in Signaling Pathways Provoked by BPAP

**Fig. 3:**
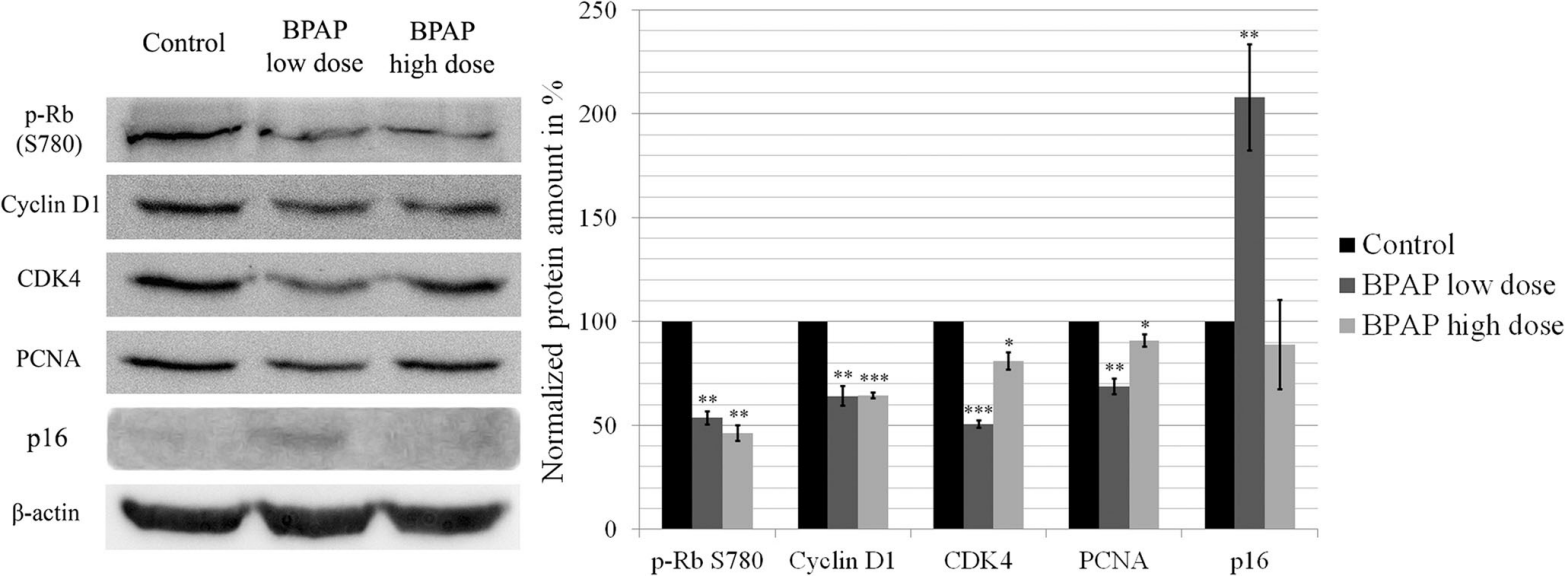
**Western blot analysis of the main cell cycle proteins.** The data are the mean ± SE of 3 experiments, *P < 0.05 and **P < 0.01

To explore the impact of BPAP on cell proliferation, regulatory proteins of the G1/S restriction point were examined (Fig. 3). The expression of Cyclin D1 significantly dropped to 50% and 60% after the low and high doses of the compound, respectively, and the changes were significant (*p* < 0.01 for low and *p* < 0.001 for high dose of BPAP). In parallel, CDK4 expression decreased by 50% and 20% after low and high doses of BPAP, respectively (p < 0.001 for low dose and *p* < 0.05 for high dose BPAP). In consequence, retinoblastoma phosphorylation on Ser780 also decreased (*p* < 0.01 for pRb regarding both doses). The decrease of PCNA level hindered the DNA synthetic activity further, down to 65% after low dose and to 85% after high dose of BPAP exposure (*p* < 0.01 and p < 0.05, respectively). In the meantime, the amount of p16^INK4^ was doubled by the low-dose (p < 0.01), whereas no effect was observable by the high-dose BPAP concentration.

**Fig. 4:**
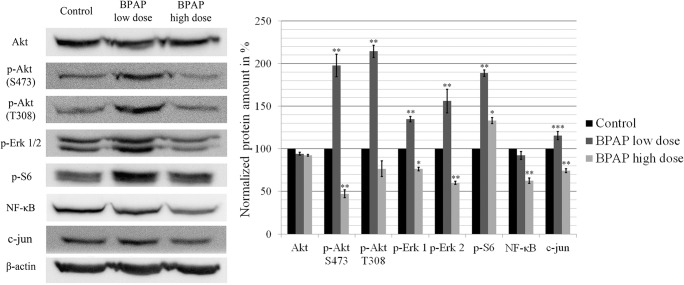
**Western blot analysis of the main signaling pathways.** The data are the mean ± SE of 3 experiments, *P < 0.05; **P < 0.01 and ***P < 0.001

Interestingly, the action of low- and high-dose BPAP proved to be different on intracellular signaling pathways (Fig. 4). Peculiarly, the low dose elevated the amount of p-Akt phosphorylated in case of both Ser473 and Thr308 by 100% (*p* < 0.01), whereas the high dose resulted a decrease by 50% (*p* < 0.01) and 25%, respectively. The same tendency was found in case of p-Erk 1/2. Low-dose increased p-Erk 1 and 2 levels by ~35% and 50% (*p* < 0.01), whereas high-dose decreased both levels by 25% and 40% (*p* < 0.05 and p < 0.01, respectively). Enhancement of mTOR signaling upon administration of both BPAP doses resulted in elevated p-S6 amounts by 89% and 33% (p < 0.01 and p < 0.05, respectively). Exposure to low and high doses of BPAP decreased NF-κB level by 10% and 40%, respectively (p < 0.01 for high dose). In addition, low-dose BPAP induced the c-jun level by 15% (*p* < 0.001), while high-dose reduced it by 30% (p < 0.01) (Fig. 4).

### BPAP against Cancer-Related Wasting

**Fig. 5:**
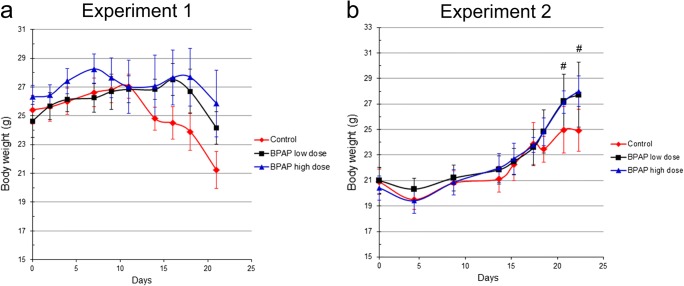
**Cancer-related wasting in FVB/N strain.** Body mass curves in Experiment 1 **(a)** and Experiment 2 **(b).** The data are the mean ± SE of the individual groups. #P < 0.05 for BPAP high dose vs. control

Treatment with either low or high doses of BPAP resulted in higher body weight compared to control animals. In Experiment 1, the loss of body mass was delayed, as the BPAP low-dose treated group had 14% higher body mass on average and the high-dose treated group 22% higher compared to the control group by the end of the study (Fig. 5a). In Experiment 2, in which case smaller tumors were inoculated, daily treatment with BPAP led to constant elevation of body weight (Fig. 5b). In contrast, the increase of body mass of tumor-bearing control animals stopped after 30 days. These results suggest that BPAP at least in part is able to compensate the body weight loss of tumor-bearing animals.

## Discussion

In the past decade, the detection of targetable somatic mutations has become essential in the treatment protocol of patients with non-small cell lung cancer [19]. The mutations discovered in the tyrosine kinase domain of EGFR and ALK gene rearrangements, as therapeutic targets have fundamentally changed the treatment of NSCLC [19, 20]. However, these alterations are responsible for only a proportion of NSCLC cases, so only conventional chemotherapies remain for patients without targetable mutations [21].

Earlier, our research group established and characterized an in vivo mouse NSCLC tumor model system, with wild type KRAS and EGFR genes, mimicking the majority of human lung adenocarcinomas [12]. It came as a surprise that when these tumors were exposed to BPAP the drug exerted a potent inhibitory effect on tumor growth. The substance was applied in a very low concentration range, with the low dose being 0.0001 mg/kg and the high dose 0.05 mg/kg bodyweight. Both doses were found to effectively hinder the proliferation of subcutaneously growing tumors. Further, the action mechanism proved to be different in case of both the signaling pathways and the cell cycle arrest at G1/S restriction point. In addition, since the effectiveness was detectable in FVB/N and SCID strains as well, the adaptive immune system is likely not involved in the mechanism [22].

**Fig. 6:**
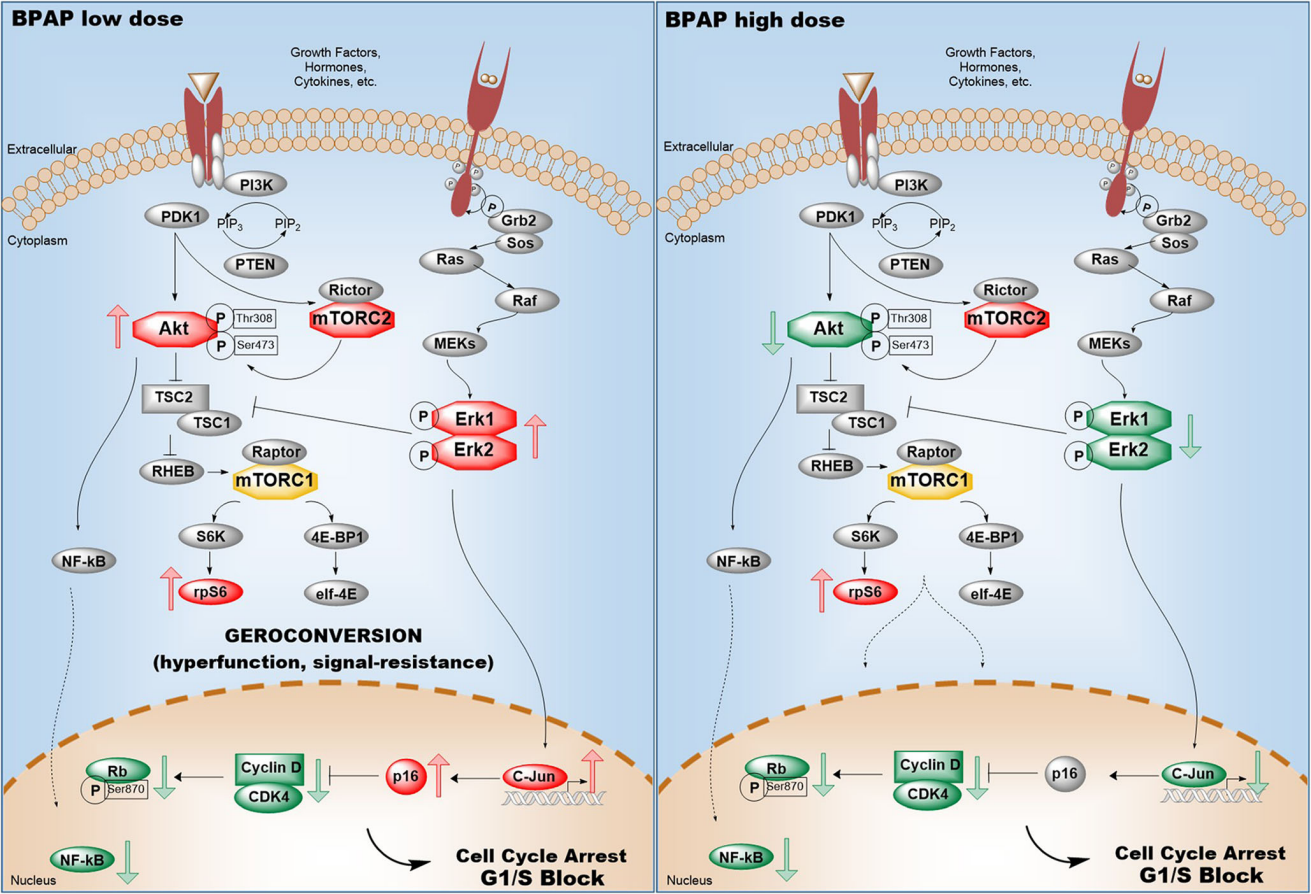
**Schematic illustration of signaling events provoked by low and high dose of BPAP.** High-dose BPAP seemed to have a direct inhibitory effect on phospho-Akt as well as on Erk 1 and 2. In contrast, the low dose provoked activation of both signaling pathways. Howsoever, BPAP increased the activity of mTOR, which was indicated by the elevated level of p-S6. Both BPAP doses inhibited the cell cycle at G1/S restriction point characterized by decreased cyclin D1 and CDK4 levels preventing the retinoblastoma from inactivation. Exposure to low dose BPAP resulted in elevated activity of the Akt/mTOR and Erk pathways together with p16^INK^-induced cell cycle arrest resulted in geroconversion

To unfold the molecular mechanism behind BPAP’s action, we focused our attention on the changes of Akt and MAPK pathways, both known to be the major downstream signaling pathways of growth factor receptors and to play important roles in NSCLC [23]. These two pathways were found to be highly activated in our model system [12, 24]. High-dose BPAP seemed to have a direct and potent inhibitory effect on both p-Akt Ser473 and Thr308 as well as on Erk 1 and 2 (Fig. 6). On the contrary, the low dose provoked activation of both signaling pathways. These findings suggest that the compound utilizes different mechanisms in low and high concentrations. Howsoever, BPAP increased the activity of mTOR, which was indicated by the elevated level of p-S6 where low dose was three times more effective. Indeed, both Akt and Erk activations (observed upon BPAP low-dose exposure) displayed excitatory effect on the mTOR pathway [25]. Inhibition of the overactivated Akt/mTOR pathway is likely an opportunity in the treatment of lung cancer [21, 26].

In spite of the different pathways provoked by low- and high-dose BPAP, the signaling events were finally culminated in cell cycle arrest. Both BPAP doses inhibited the cell cycle at G1/S restriction point since the levels of cyclin D1 and CDK4 became decreased, thus preventing the retinoblastoma from inactivation. The lower PCNA levels observable upon BPAP treatment also point to inhibition of cell proliferation.

In case of low-dose BPAP, the highly stimulated signaling pathways in contrast to the ineffective G1 phase and hindered tumor growth called attention to the mechanism of geroconversion. In this hyper-mitogenic type of arrest, the cell cycle is suppressed by CDK inhibitors (such as p21^WAF1/CIP1^ or p16^INK4^), but mTOR and MAPK pathways are still active pushing the cell to become hyperthophic, hyper-active and hyper-functional. Activation of mTOR in the presence of cell cycle arrest induces senescent morphology and the loss of ability to proliferate [27–29]. The fact that we observed an increased amount of p16 accompanied by a high level of mTOR activity supports our presumption that low-dose BPAP provoked geroconversion upon treatment (Fig. 6). Interestingly, similar effect was observed upon exposure to PARP inhibitor in case of liposarcoma and other tumors, however, upregulation of p21^WAF1/CIP1^ and proteosomal degradation of MDM2 were accompanied by downregulation of CDK4 [30, 31].

The question is, what is the mechanism of p16 upregulation? A recent publication provides evidence that the AP-1 factor c-jun is able to interfere with tumorigenesis by protecting the promoter region of p16^INK4a^ from methylation, acting as a “bodyguard” after oncogenic transformation [32]. As we detected strong Erk1/2 activation, with subsequent upregulation of c-jun upon low dose BPAP exposure, it is highly conceivable that c-jun is implicated in the upregulation of p16^INK^.

In contrast with the results observed after low-dose BPAP, the high dose decreased the level of c-jun. C-jun is found to be elevated in 30% of NSCLC cases and cannot be found in the normal cells of airways [33, 34].

Despite the similar outcome, our data show that BPAP in low and high doses utilizes different mechanisms of action, since the latter exerts direct inhibition of the main signaling pathways (Fig. 6). A further question therefore is, what resulted the activation of p-S6 after BPAP high-dose administration? [35, 36].

Cancer cachexia is a terminal state in which patients lose their body fat and muscle weight rapidly as compared with immune system failures and metabolic dysfunctions [37]. Both low- and high-dose BPAP exposure had a beneficial effect on cachexia. High-dose BPAP showed better protection, saving 22% more of the animal’s body mass. NF-κB is a key regulator of the events implicated in the development of cachexia [38]. NF-κB has a role also in chemotherapy resistance, thus its inhibition may potentiate the efficacy of the therapy [39].

## Conclusions

BPAP is a potent enhancer substance related to certain signaling events in the central nervous system. In a longevity study, it was discovered that the substance is also effective against certain types of cancers, showing the most promising results in case of lung cancer. In our study, the effects of two different doses of BPAP were studied in a newly developed EGFR wild type mouse lung adenocarcinoma xenograft. Both doses showed good results in tumor and cachexia inhibition, although seemingly having different mechanisms of action. High dose of BPAP showed direct inhibition on the main signaling pathways, whereas the low dose seemed to initiate geroconversion. Elevated activity of the mTOR pathway together with p16^INK^-induced cell cycle arrest resulted in geroconversion, which is a senescent state accompanied by loss of cell proliferation.

In view of the low toxicity and confirmed antitumor effect of BPAP in case of experimental lung adenocarcinoma, this novel compound deserves further attention.

## Electronic supplementary material

ESM 1 (DOC 41 kb)

## Abbreviation

Akt: protein kinase B
ALK: anaplastic lymphoma kinase
BPAP: (2R)-1-(1-Benzofuran-2-yl)-N-propylpentan-2-amine
CDK4: cyclin-dependent kinase 4
EDTA: ethylene diamine tetraacetic acid
EGFR: epidermal growth factor receptor
HRP: horseradish peroxidase
KRAS: Kirsten Rat Sarcoma virus oncogene
MAO-B: monoamine-oxidase B
MDM2: mouse double minute 2 homolog
mTOR: mechanistic target of rapamycin
NSCLC: non-small cell lung cancer
PARP: poly ADP ribose polymerase
PCNA: ploriferating cell nuclear antigen
PVDF: polyvinylidene difluoride
Rb: retinoblastoma
SCID: severe combined immunodeficiency
TBS: Tris-buffered saline
