# Supplementary material for: Inhibitory Effect of (2R)-1-(1-Benzofuran-2-yl)-N-propylpentan-2-amine on Lung Adenocarcinoma
Source: Pathol Oncol Res. 2019 Feb 8;26(2):727–34. doi: 10.1007/s12253-019-00603-6 (PMC7242259; doi:10.1007/s12253-019-00603-6)
Supplement: Supplementary file 1 — (DOC 41 kb) [file 12253_2019_603_MOESM1_ESM.doc]

**Table 1. Antibodies used in the present study.**

| Primary antibody | Manufacturer | Catalog number | Source | Dilution |
| --- | --- | --- | --- | --- |
| Akt | Cell Signaling Technology, Danvers, MA | #4691 | rabbit | 1:1000 |
| p-Akt (Thr308) | Cell Signaling Technology | #2965 | rabbit | 1:1000 |
| p-Akt (Ser473) | Cell Signaling Technology | #4058 | rabbit | 1:1000 |
| p-Erk1/2 | Cell Signaling Technology | #4370 | rabbit | 1:1000 |
| p-S6 | Cell Signaling Technology | #2211 | rabbit | 1:1000 |
| CDK4 | Neomarkers, Fremont, California, US | #MS-616 | mouse | 1:250 |
| PCNA | Atlas antibodies, Stockholm, Sweden | HPA030522 | rabbit | 1:1000 |
| Cyclin D1 | Neomarkers, Fremont, California, US | MA5-14512 | rabbit | 1:250 |
| p-Rb S780 | Cell Signaling Technology | #9307 | rabbit | 1:250 |
| NF-κB | Cell Signaling Technology | #8242 | rabbit | 1:1000 |
| p16 | LabVision-Thermo Fisher Scientific, Waltham, US | MA5-17093 | mouse | 1:250 |
| c-jun | Cell Signaling  Technology | #9165 | rabbit | 1:1000 |
| β-actin | Sigma-Aldrich, St. Louis, MO | A2228 | mouse | 1:5000 |
